# Engineering a vitamin B_12_ high-throughput screening system by riboswitch sensor in *Sinorhizobium meliloti*

**DOI:** 10.1186/s12896-018-0441-2

**Published:** 2018-05-11

**Authors:** Yingying Cai, Miaomiao Xia, Huina Dong, Yuan Qian, Tongcun Zhang, Beiwei Zhu, Jinchuan Wu, Dawei Zhang

**Affiliations:** 1 Tianjin Institute of Industrial Biotechnology, Chinese Academy of Sciences, 32 West 7th Avenue, Tianjin Airport Economic Area, Tianjin, 300308 China; 2 Key Laboratory of Systems Microbial Biotechnology, Chinese Academy of Sciences, 32 West 7th Avenue, Tianjin Airport Economic Area, Tianjin, 300308 China; 3 College of Biotechnology, Tianjin University of Science & Technology, No. 29, thirteenth Avenue Binhai District, Tianjin, 300457 China; 4 School of Food Science and Technology, Dalian Polytechnic University, National Engineering Research Center of Seafood, Dalian, 116034 People’s Republic of China; 5 Industrial Biotechnology Division, Institute of Chemical and Engineering Sciences, 1 Pesek Road, Jurong Island, 627833 Singapore

**Keywords:** Vitamin B_12_, Cobalamin, *Sinorhizobium meliloti*, Riboswitch, High-throughputScreening

## Abstract

**Background:**

As a very important coenzyme in the cell metabolism, Vitamin B_12_ (cobalamin, VB_12_) has been widely used in food and medicine fields. The complete biosynthesis of VB_12_ requires approximately 30 genes, but overexpression of these genes did not result in expected increase of VB_12_ production. High-yield VB_12_-producing strains are usually obtained by mutagenesis treatments, thus developing an efficient screening approach is urgently needed.

**Result:**

By the help of engineered strains with varied capacities of VB_12_ production, a riboswitch library was constructed and screened, and the *btuB* element from *Salmonella typhimurium* was identified as the best regulatory device. A flow cytometry high-throughput screening system was developed based on the *btuB* riboswitch with high efficiency to identify positive mutants. Mutation of *Sinorhizobium meliloti* (*S. meliloti*) was optimized using the novel mutation technique of atmospheric and room temperature plasma (ARTP). Finally, the mutant *S. meliloti MC5–2* was obtained and considered as a candidate for industrial applications. After 7 d’s cultivation on a rotary shaker at 30 °C, the VB_12_ titer of *S. meliloti MC5–2* reached 156 ± 4.2 mg/L, which was 21.9% higher than that of the wild type strain *S. meliloti* 320 (128 ± 3.2 mg/L). The genome of *S. meliloti* MC5–2 was sequenced, and gene mutations were identified and analyzed.

**Conclusion:**

To our knowledge, it is the first time that a riboswitch element was used in *S. meliloti.* The flow cytometry high-throughput screening system was successfully developed and a high-yield VB_12_ producing strain was obtained. The identified and analyzed gene mutations gave useful information for developing high-yield strains by metabolic engineering. Overall, this work provides a useful high-throughput screening method for developing high VB_12_-yield strains.

**Electronic supplementary material:**

The online version of this article (10.1186/s12896-018-0441-2) contains supplementary material, which is available to authorized users.

## Background

Vitamin B_12_ (cobalamin, VB_12_) is one of the most structurally complex natural Vitamins. VB_12_ is only produced by some bacteria and archaea and it requires more than 30 enzymes for the de novo synthesis of VB_12_ [1, 2]. VB_12_ plays an important role as an essential co-factor in various biochemical processes such as DNA synthesis, fatty acid synthesis, amino acid metabolism and energy production [3, 4]. VB_12_ has been widely used in food and medicine fields [5], and the global demand for VB_12_ in these applications has been growing rapidly [6, 7]. A considerable number of enhancement and development of natural products mainly rely on mutagenesis and screening strategies [8], while targeted engineering strategies by gene overexpression or deletion do not always generate desired results due to the complexity of highly regulated metabolic networks.

In order to screen libraries with high genetic diversity, a key problem is developing smart screening methods. Traditional methods suffer from low dynamic range, low throughput and unwanted sensitivity to variable experimental parameters [9]. Fluorescence-activated cell sorting (FACS) allows ultrahigh throughput in cell sorting and requires a sortable signal output by biosensors in individual cells that respond to various intracellular concentrations of the target molecule with a correlation [10]. Developing a biosensor sensitive to VB_12_ concentration is thus very important.

Riboswitches are small structured mRNA elements that regulate gene expression and translation in response to specific small molecule ligands [11]. They act as relatively simple and direct metabolite sensors that are capable of both molecular recognition and transducing this information by altering protein expression [12–14]. Natural riboswitches are found with the highest frequency in the 5’-UTR of bacterial mRNAs, where they regulate the expression of downstream genes through structural changes undergone in response to the binding of a specific target molecule.

**Fig. 1:**
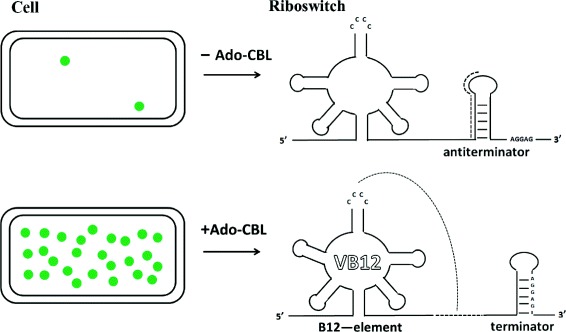
Mechanism of riboswitch based intracellular vitamin B_12_. In the simplified example cells containing a very low concentration or none of the VB_12_ (green circle), the ribosome binding site (RBS) is unstructured. Under a higher concentration of target metabolite, the aptamer domain undergoes restructuring forming a hairloop to attenuate gene expression

VB_12_ riboswitch has been found to widely regulate VB_12_ biosynthesis in bacteria [15]. Expression of cob operon in cobalamin biosynthetic pathway and transporter encoding gene *btuB* are repressed by the presence of vitamin B12, in particular. The VB_12_ riboswitch consists of a binding region (aptamer domain) and an expression platform that modulates gene expression [16, 17]. Upon ligand binding, the aptamer region undergoes a conformational change that is interpreted by the expression platform (Fig. 1), resulting in repressed gene expression [18, 19]. Comparative analysis of the genes and regulatory regions from different bacterial genomes is a powerful approach allowing the identification of regulatory patterns [20]. Using this approach, Vitreschak et al. have summarized approximately 200 VB_12_-elements from 67 bacterial genomes [21], providing candidates for biosens or development.

In this study, we screened a sensitive riboswitch to sense the amount of intracellular VB_12_ concentration of single cells by FACS and developed a VB_12_ high-throughput screening system. Using this novel VB_12_ screening system, high yield VB_12_ producing strains were screened and identified from a mutant library of *S. meliloti* generated by ARTP. Moreover, genomic analysis of the high yield VB_12_ producing strain allowed us to give insight into the mechanism of VB_12_ regulation pathways which would guide future study.

## Methods

### Strains and culture conditions

The initial strain, *S. meliloti 320*, was stored in our laboratory [22]. *S. meliloti 320* was grown in LB medium supplemented with MgCl_2_ (2.5 mM) and CaCl_2_ (2.5 mM) (LB/MC medium), or minimal M9 medium supplemented with 1 μg/mL biotin and 10 ng/mL CoCl_2_, as well as 0.1% sucrose for liquid medium or 0.2% sucrose for solid medium (unless indicated otherwise), respectively, (named M9/sucrose) [23]. And the strain was grown at 30 °C with shaking at 200 rpm. Antibiotics were used as follows: spectinomycin (Sm; 200 μg/mL for *E. coli* and 600 μg/mL for *S. meliloti*), gentamicin (Gm; 10 μg/mL for *E. coli*, 20 μg/mL for *S. meliloti*), and kanamycin (Km; 50 μg/mL for *E. coli* and 100 μg/mL for *S. meliloti*).

### Genetic techniques

For construction of *Sm320-N8* strain, genetic deletions in *S. meliloti* 320 genome were generated by homologous recombination and allelic replacement. Two fragments of cobI-left and -right flanking DNA sequences (Additional file 1: Table S1) were ligated by overlap extension polymerase chain reaction (PCR), and the PCR product was cloned into a suicide vector pJQ200SK carrying *sacB* and gentamicin resistance gene. The construct was introduced into *S. melilothi* by triparental mating with help of the mobilizing strain MT616 [24] carrying pRK600, and the culture was plated onto LB/MC-agar containing gentamicin. A second reciprocal recombination event was selected on LB/MC-agar containing 5% sucrose, and then isolates with the desired deletions were subsequently screened by PCR verification in which primers hybridizing outside the cloned flanking sequences were used to amplify the deletion region.

### Riboswitch sensor construction and validation

To construct riboswitch sensor plasmids, the promoter PmelA was amplified from pSymB megaplasmid, the approximately 200 bp VB_12_ riboswitch sequence containing a riboswitch and RBS from different strains were obtained by artificial synthesis (riboswitch sequences were provided in Additional file 1: Table S2), and the genes were amplified by PCR. Fragments containing PmelA promoter, VB_12_ riboswitch sequence and GFP reporter gene were fused by PCR. Next, the pBHR1 plasmid and the fused PCR fragments were ligated by Gibson assembly at 50 °C for 1 h, and then cloned in *E. coli* strain *DH5α*. Detailed constructions of the riboswitch-containing plasmids are shown in Additional file 1: Figure S2 and Additional file 1: Table S2. The plasmids were then separately transferred into *Sm320*, *Sm320-N8* and *Sm320-L6* (engineered mutants with different production capacities) by triparental mating. *S. meliloti* containing P_melA_-riboswitch-gfp plasmid was grown at 30 °C for 12 h, and then transferred into shake flask fermentation with 10% (v/v) of inoculation for 3 days. The samples were harvested with centrifuging at 4000 g for 8 min, followed by washing in PBS for three times. The cells were collected to measure their GFP fluorescence by flow cytometry [9].

The flow cytometry (MoFlo XDP, Beckman, USA) was performed with a 488-nm excitation laser and FL1, respectively. The data were analyzed with Summit software. Eight riboswitches were selected based on the sequence reported by Vitreschak et al. This method was also used to characterize the other 7 strains. The strains and plasmids used in this study are presented in Table 1 and sequences of primers are listed in Table S1 in the Additional file 1.

**Table 1:** Strains and plasmids used and constructed in this study

Strain or plasmid	Relevant characteristics	Sources of reference
Strains		
Sm320	*Sinorhizobium meliloti 320,* VB_12_ producing strain.	[22]
Sm320-L6	*Sm320* derivative, carrying plasmid pBR-OhemE to reduce production capacity	This study
Sm320-N8	*Sm320* derivative, knocking out *cobI* gene	This study
Sm320-Mybi	*Sm320* derivative, carrying plasmid pBR- SYB-lacI and plasmid pMB-gfp was used in the ARTP treat	This study
MT616	MT607/pRK600; *Escherichia coli* helper strain for mobilizing RK2/RP4-derived plasmids	[24]
Plasmid		
pJQ200sk	Vector for construction of deletions, *sacB*,Gm^R^	[31]
pMB393	Broad-host-range vector, P_tauA_ promoter,Amp^R^	[32]
pBHR1	Broad-host-range vector, Derivative of PBBR122,Km^R^	This lab
pBR- EC-butB	pBHR1 plasmid carrying EC-butB riboswitch were cloned in front of a *gfp* reporter gene driven by the constitutive promoter P_melA_	This study
pBR- PF-cbiB	pBHR1 plasmid carrying PF-cbiB riboswitch were cloned in front of a *gfp* reporter gene driven by the constitutive promoter P_melA_	This study
pBR- SM-bluB	pBHR1 plasmid carrying SM-bluB riboswitch were cloned in front of a *gfp* reporter gene driven by the constitutive promoter P_melA_	This study
pBR- SM-cobU	pBHR1 plasmid carrying SM-cobU riboswitch were cloned in front of a *gfp* reporter gene driven by the constitutive promoter P_melA_	This study
pBR- MLO-bluB	pBHR1 plasmid carrying MLO-bluB riboswitch were cloned in front of a *gfp* reporter gene driven by the constitutive promoter P_melA_	This study
pBR- MLO-metE	pBHR1 plasmid carrying MLO-metE riboswitch were cloned in front of a *gfp* reporter gene driven by the constitutive promoter P_melA_	This study
pBR- SY-btuB	pBHR1 plasmid carrying SY-btuB riboswitch were cloned in front of a *gfp* reporter gene driven by the constitutive promoter P_melA_	This study
pBR- BI-cbiW	pBHR1 plasmid carrying BI-cbiW riboswitch were cloned in front of a *gfp* reporter gene driven by the constitutive promoter P_melA_	This study
pBR- SYB-lacI	pBHR1 plasmid carrying SY-btuB riboswitch were cloned in front of a *lacI* reporter	This study
pMB-gfp	pMB393 containing *lacO* and carrying *gfp* reporter	This study
pBR-OhemE	pBHR1 plasmid carrying *hemE* gene to reduce production capacity	This study

### Mutagenesis by ARTP and determination of optimal treatment time

Pure helium was used as the plasma working gas for ARTP mutation and the operating parameters were as follows: (1) the radio frequency power input was 100 W; (2) the distance between the plasma torch nozzle exit and the sample plate was 4 mm; and (3) the temperature of the plasma jet was below 30 °C [25]. Under these operating conditions, the ARTP mutagenesis dosage was only dependent on the treatment time.

To determine the optimal treatment time, *S. meliloti* was cultured for 72 h (mid-log phase) and then subjected to ARTP treatment. Briefly, 10 μL culture (1 × 10^8^ cells/mL) was treated with the helium-based ARTP for 15, 30, 60, 75, 90 and 120 s. Lethality rate was calculated for determining the appropriate treatment time. The culture without treatment was used as a control. After each treatment, the steel plate was eluted with sterile distilled water into new tubes and properly diluted, incubated on fermentation medium at 30 °C for 1 h, and then cultured on solid medium with 600 μg/mL spectinomycin at 30 °C for 4 days. The lethality rate was determined as follows:

$$ \mathrm{Lethality}\ \mathrm{rate}\ \left(\%\right)=\frac{\mathrm{control}\ \mathrm{colonies}-\mathrm{survival}\ \mathrm{colonies}}{\mathrm{control}\ \mathrm{colonies}}\times 100\% $$

### Whole genome sequencing

The genomic DNA of strain *S. meliloti 320* was extracted from bacterial cells grown overnight in LB/MC medium at 30 °C and 200 rpm. The DNA was extracted using OMEGA Bacterial DNA kits (Omega bio-tek, Feyou Biotechnology, China) following the manufacturer’s protocol [26, 27]. To verify DNA quality, PCR amplifications were performed in a final volume of 50 μL, containing 10 μM each primer, 1.25 units of Q5 High-Fidelity DNA polymerase (New England Biolabs, USA), dNTP mixture (250 μM each dNTP), and approximately 50 ng of template DNA. The PCR amplification conditions consisted of an initial denaturation for 10 min at 94 °C, followed by 30 cycles of denaturation at 94 °C for 30 s, annealing at 55 °C for 30 s, and extension at 72 °C for 1 min with a final extension of 10 min at 72 °C. The quality and quantity of DNA were analyzed using 1% agarose gel electrophoresis and spectrophotometric method, respectively. The verified genomic DNA was selected for further analysis.

### Analytical methods

Biomass was determined with a spectrophotometer at 600 nm. The sample for measuring cell concentrations was obtained as follows: 1 mL of broth was centrifuged at 10,000×g for 10 min, the supernatant was discarded, and the cell pellets were resuspended in 1 mL of 0.85% NaCl buffer. The concentrations of the principal fermentation products were determined by high performance liquid chromatography (HPLC) [28]. The sample (25 mL) was mixed with 2.5 mL of NaNO_2_ 8% (*w*/*v*) and 2.5 mL of glacial acetic acid. The mixtures were boiled for 30 min, and then centrifuged. The supernatants were filtrated through a 0.22-μm membrane filter. Then, 20 μl of NaCN 10% (w/v) was added to 1 mL of the aqueous phase. 20 μL of the final sample was injected into an Agilent 1260 HPLC with a 5C18-250A column (Agilent, 4.6 mm id × 250 mm, 5 μm) thermostated at 35 °C. The mobile phase consists of water and acetonitrile (70:30 [v/v]) at 0.8 mL/min [22]. Samples were detected at 361 nm. All values presented in this study were averages of at least three independent trials.

### Preparation of cell extracts and detection of the amount of intracellular VB_12_

Centrifugation was used to sediment 500 μL of cell suspension through 100 μL silicone oil on the bottom of the tube. Cell pellets were then resuspended by 200 μL perchloric acid (20%, v/v) by gentle vortexing after disruption by sonication. After breaking up the cells, extracts were neutralized by addition of 200 μL Na_2_CO_3_ and the membrane materials and denatured proteins were removed by centrifugation. Then, 100 μL of NaNO_2_ 8% (*w*/*v*) and 100 μL of glacial acetic acid were added to 1 mL of aqueous phase, and the mixture was boiled for 30 min, centrifuged and the supernatant was filtrated through a filter membrane (0.22 μm), and then 2 μl of NaCN 10% (w/v) was added to 100 μl of the aqueous phase. The samples were stored in − 20 °C for VB_12_ analysis by HPLC. The amount of intracellular VB_12_ was calculated as the mill grams of intracellular VB_12_ per liter of culture.

### Fermentations

The fermentation medium contained (per liter of deionized water): sucrose 60 g/L; corn steep liquor (CSL) 20 g/L; betaine 6 g/L; ammonium sulfate 2 g/L; potassium dihydrogen phosphate 2.6 g/L; cobalt chloride 0.06 g/L; and 0.1 g/L DMB was added to the medium. The pH before autoclaving was adjusted to 7.0–7.4. The stock sugar solution was autoclaved separately before mixing with the rest of the medium.

For cultivating VB_12_-producing strains in microtiter plates, colonies were cultivated in 100 μL LB/MC medium overnight using 96-well microtiter plates (Corning Costar 3930, square U-bottom, 350 μL) and then 10% (v/v) inocula was transferred to 2 mL fermentation medium using 24-well deep microtiter plates (Corning Costar 3524, square U-bottom, 5 mL) at 30 °C, 700 rpm, 80% humidity in a Microtron shaker (Infors, China).

For shake flask fermentation, the seed culture was grown at 30 °C for 12 h and was used to provide 10% (v/v) inocula for fermentation. Experiments were conducted in 250 mL Erlenmeyer flasks containing 30 mL of each medium at 30 °C at an agitation rate of 200 rpm in rotary shakers for 7 days. The samples were used to determine cell mass (OD600 nm), residual sugars every 24 h, and product at 7 days.

## Results

### Engineered mutants with different production capacities of VB_12_

**Fig. 2:**
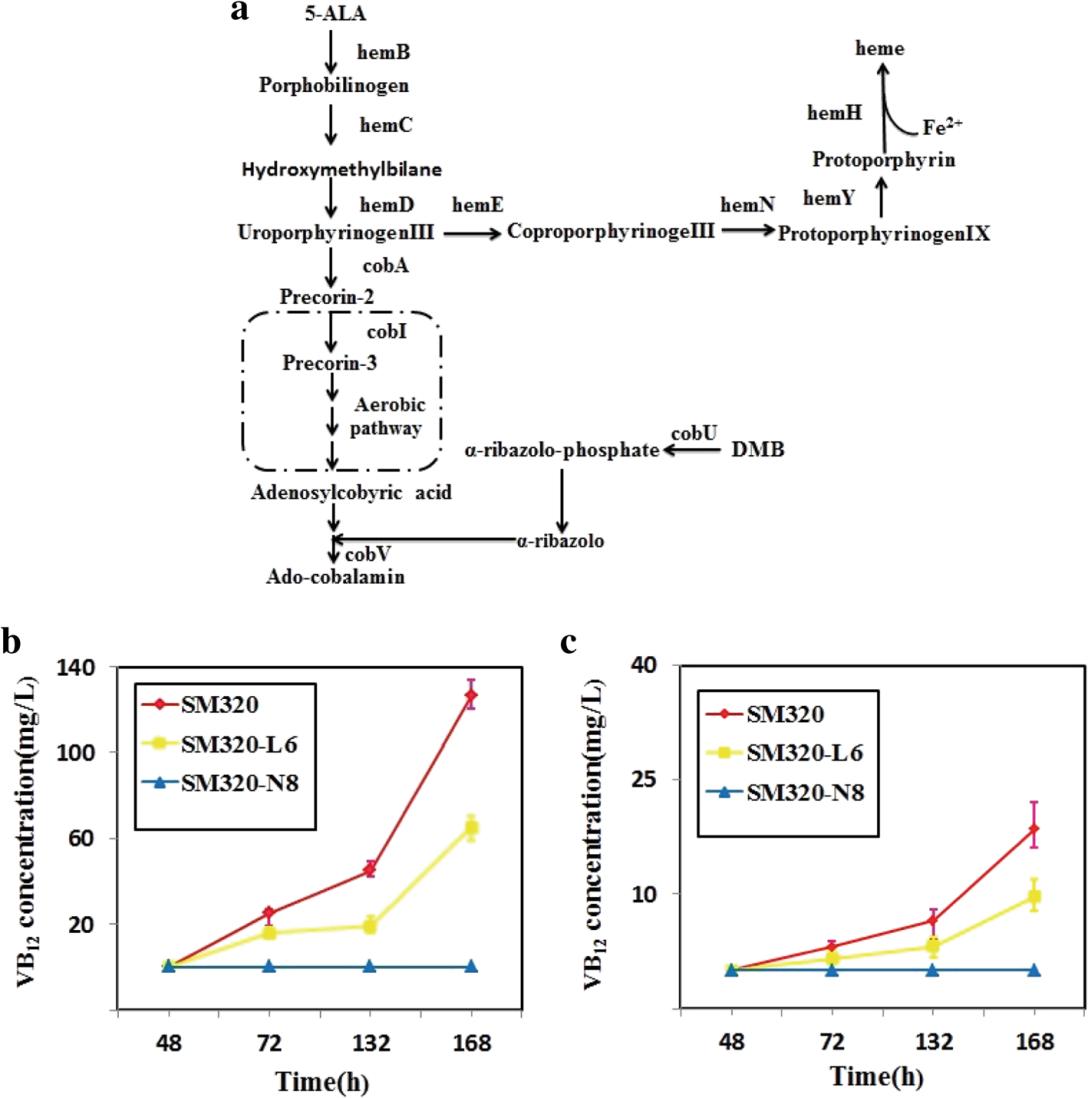
Constructed engineered mutants with different production capacities. **a** Oxygen-dependent VB_12_ biosynthetic pathway in *S. meliloti*. The branched biosynthesis of heme is also depicted. **b** The concentration of VB_12_ in producer strains in fermentation broth was determined by HPLC. Different colors represent the different strains. Values shown are HPLC-determined and are the means of three independent fermentation cultures for each strain. **c** the amount of intracellular VB_12_ in recombinant strains in (**b**)

To evaluate the dose-response of riboswitches on transcriptional regulation under variable the amount of intracellular VB_12_ levels, we engineered mutants of the original *S. meliloti* production strain *Sm320* with different production capacities. The complete VB_12_ biosynthetic pathway contains approximately 30 genes (Fig. 2a) and deleting any of them will prevent VB_12_ biosynthesis. Strain *Sm320-N8* with an in-frame deletion of essential VB_12_ biosynthetic gene *cobI* was constructed as a negative control strain that does not produce VB_12_. To construct mutant with low production capacity, we overexpressed gene *hemE* encoding the key enzyme in the heme branch pathway to reduce VB_12_ biosynthesis. This strain was named *Sm320-L6*. The VB_12_ concentration of the original strain *Sm320* and the two derivatives, *Sm320-N8* and *Sm320-L6*, were measured by HPLC. The total amounts of VB_12_ produced by strains *Sm320-N8*, *Sm320-L6* and *Sm320* were 0, 65.21 and 126.28 mg/L with 7d’s cultivation, respectively, the amounts of intracellular VB_12_ of strains *Sm320-N8*, *Sm320-L6* and *Sm320* were 0, 9.65 and 18.5 mg/L (Fig. 2b, c). The details of cell growth are provided in Additional file 1: Figure S1. It was important to note that the amounts of intracellular VB_12_ was used to indicate the intracellular VB_12_ level, and but was not the real intracellular VB_12_ concentration.

### Construction of riboswitch-based VB_12_ sensors

**Fig. 3:**
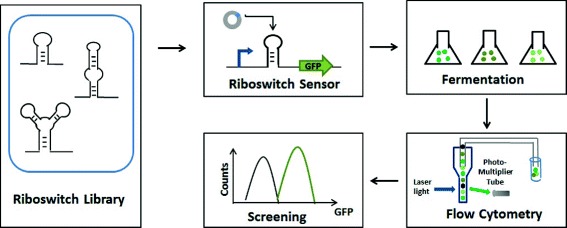
Flow diagram of constructed VB_12_ riboswitches. The ideal phenotypes were selected from a VB_12_ riboswitch library. VB_12_ sensor constructs were made that contained a predicted riboswitch and a GFP reporter. The fermentation broth was collected and analyzed by Fluorescence Activated Cell Sorting

To identify a sensitive element that allows efficient screening of positive mutants with high VB_12_ production, eight different potential riboswitches (Table 2) were selected according to the criteria described above, plasmids containing VB_12_-dependent riboswitches coupled to a GFP reporter were separately transformed into *Sm320*, *Sm320-L6* and *Sm320-N8*, then the expression levels of GFP under the control of the eight riboswitches were compared. The strains containing the plasmids were grown at 30 °C for 12 h and were used to provide shake flask fermentation for 3 days with 10% (v/v) inoculant and then tested for GFP expression by flow cytometry (Fig. 3).

**Table 2:** Conserved RNA elements upstream of some B_12_-regulated genes

Genome abbreviations	The start codon	Proximal downstream genes	Coding of enzyme protein
CBL biosynthesis			
Propionibacterim freudenreichii (PF)	285	*cbiB*	VB_12_ biosynthesis protein
Bacillus megaterium(BI)	266	*cbiW*	VB_12_ biosynthesis protein
Mesorhizobium loti(MLO)	233	*bluB*	5,6-dimethylbenzimidazole synthase
Sinorhizobium meliloti(SM)	251	*bluB*	5,6-dimethylbenzimidazole synthase
Sinorhizobium meliloti(SM)	227	*cobU*	adenosylcobinamide kinase
Vitamin B12 transport			
Escherichia coli(EC)	248	*btuB*	VB_12_ transporter BtuB
Salmonella typhimurium(SY)	252	*btuB*	VB_12_ transporter BtuB
B12-dependent or alternative metabolic pathways			
Mesorhizobium loti(MLO)	309	*metE*	5-methyltetrahydropteroyltriglutamate--homocysteine methyltransferase

**Fig. 4:**
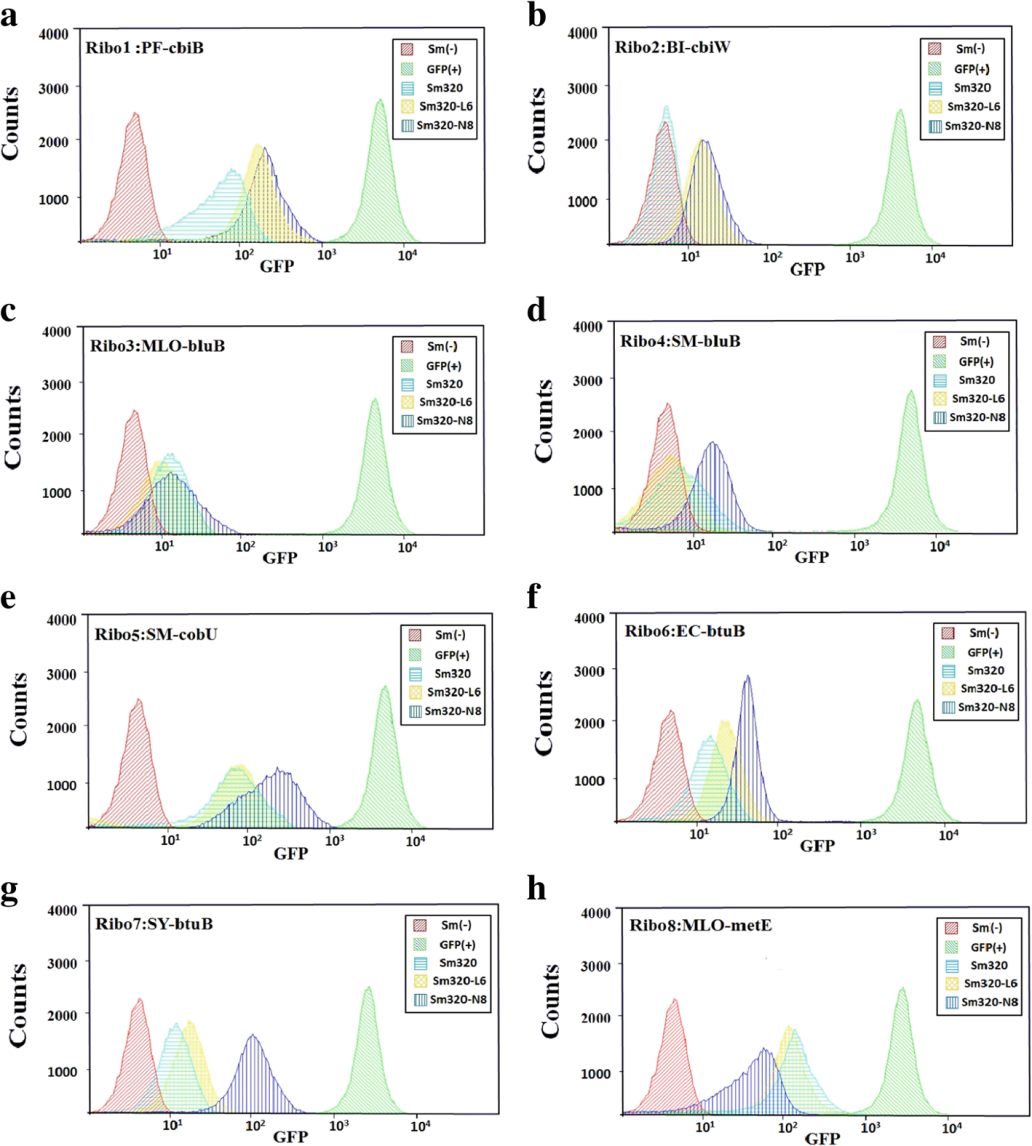
Validation and selection of a sensitive element that allows efficient identification of positive mutants. Fluorescence-activated cell sorting results of engineered VB_12_ producing strains with different production capacity (*Sm320-N8*, *Sm320-L6,* and *Sm320*) were tested separately with eight riboswitches. Fig. **a**-**h** exhibited *S. meliloti *strains carrying different riboswitches, and the genome abbreviations of eight different potential riboswitches were marked in the top left corner of the figures

Figure 4 shows the GFP fluorescence of different *S. meliloti* strains carrying different riboswitches. The strains containing riboswitches shown in Fig. 4-b, c, d, e and h exhibited a similar fluorescence intensity, although they could produce different levels of VB_12_. Fig. 4-g shows that the fluorescence intensity of different *S. meliloti* strains containing SY-btuB-gfp plasmid were sensitive to the increasing amount of intracellular VB_12,_ while the other strains in Fig. 4-a, f, g exhibited a negative correlation between their fluorescence intensity and the amounts of intracellular VB_12_. The results indicated that some of these predicted riboswitches indeed responded to the concentrations of VB_12_ in *S. meliloti*. Thus, we further examined the feasibility of using the SY-btuB riboswitch as a sensitive sensor for VB_12_ concentration.

### Examination of the SY-btuB riboswitch sensor

**Fig. 5:**
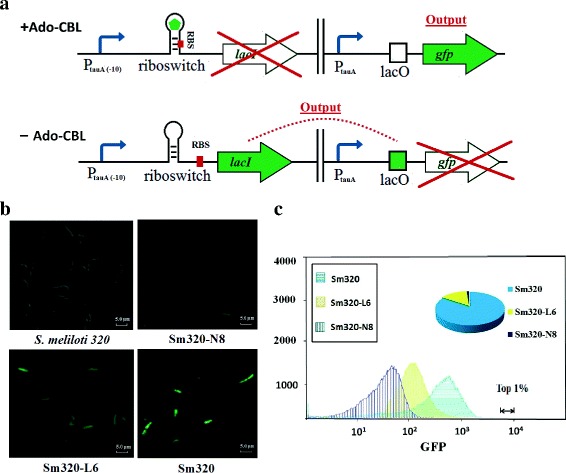
Validation of feasibility of riboswitch SY-btuB in different performances. **a** Mechanism of VB_12_ dependent riboswitch SY-btuB double plasmid work mode. VB_12_ directly binds to the riboswitch region, producing a conformational change in the secondary structure of mRNA, and thus inhibiting *lacI* gene expression and causing the *gfp* reporter to show a fluorescent signal. For cells containing a very low concentration of VB_12_, the ribosome binding site (RBS) is unstructured. This allows for efficient translation of *lacI* gene and then *lacI* and can bind *lacO* and repress *gfp* expression. **b** The microscopic image of *S. meliloti 320* and fluorescence microscopic images of *Sm320, Sm320-L6* and *Sm320-N8* cells carrying SY-btuB sensor plasmid, respectively. **c** FACS (Fluorescence Activated Cell Sorting) results of three strains mixed with equal optical density. Strains exhibiting top 1% of the fluorescence values were selected by FCM. The screened strains were then distinguished the selected colonies by Summit 5.2 analysis

We attempted to demonstrate the feasibility of the sensor for screening hyperproducing strains from a library. As SY-btuB VB_12_ riboswitch is a repressive type of selector, the single *gfp* reporter gene was substituted with a “dual-repression” mode that contained a *lacI* repressor protein and a *gfp* reporter gene (Fig. 5a). We tested this reconstructed sensor in strains *Sm320*, *Sm320-L6* and *Sm320-N8*. A positive correlation between the GFP fluorescence and the VB_12_ productivity of the strains was showed by flow cytometry (Fig. 5c).

In order to verify the response behavior of the new sensor system to the amount of intracellular VB_12_ level, GFP fluorescence was observed by fluorescence microscope (DM5000B, Leica, Germany). A very weak fluorescence value was detected for the samples without VB_12_, and there was a corresponding increase in GFP fluorescence value along with the increase amount of intracellular VB_12_ (Fig. 5b). This result indicated that this sensor could work in different strains to sense the different levels of the amount of intracellular VB_12_ by producing corresponding levels of fluorescence intensity. Moreover, a corresponding rise in GFP fluorescence values of *Sm320-N8* strains carrying SY-btuB sensor double plasmids could be observed by increasing VB_12_ concentration in the medium (Additional file 1: Figure S3).

Next, we simulated a screening process to verify the reliability of the sensor-reporter. The three strains with different production capacities were mixed together at equal optical density and the mixture of strains was used for screening with FCAS. Colonies exhibiting the top 1% of fluorescence values were analyzed to evaluate the sorting performance of the SY-btuB sensor double plasmid (Summit 5.2 analysis). As expected, nearly 84% of sorted cells were identified as the high-producing *Sm320* strain (Fig. 5c). Overall, *Sm320-Mybi* represented the best regulatory device, and the strain *Sm320-Mybi* was used in the subsequent experiments.

### High throughput screening of mutants using riboswitch

ARTP-irradiation was used to induce random mutations, and high VB_12_-producing mutants were selected. Growing cells were treated with helium-based ARTP for 15, 30, 60, 75, 90 and 120 s, respectively. The lethality rate was over 80% when the exposure time exceeded 60 s, and there was no cell survival after 120 s treatment (Fig. S4). Therefore, the optimal exposure time used in subsequent studies was set at 60 s with lethality ranged from 80 to 85%.

We then attempted to screen hyperproducing strains from a library of variant strains using the engineered riboswitch. After treatment of ARTP, selection was carried out using the “dual-repression” SY-btuB riboswitch. Colonies exhibiting the top 0.1% of fluorescence signals were selected from the genetic library. The wild type strain and the mutants were cultured in LB/MC medium in a 96-well microtiter plate at 30 °C for 12 h and then transferred to fresh medium for fermentation in 24-well deep plate at 30 °C with shaking for 7 days.

**Fig. 6:**
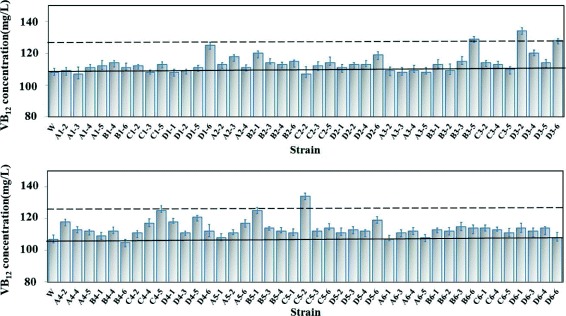
VB_12_ concentration of *Sm320* mutants obtained with ARTP treatments. “W” indicates the wild-type strain of *S. meliloti* with a VB_12_ content of 108 mg/L in a 24-well deep plate at 30 °C for 7 d. The solid line indicates the original strain, and the dotted line indicates the position where the VB_12_ content increased 10% compared with the original strain. Error bars show the means of three independent fermentation cultures for each strain by HPLC analysis

After 3 rounds of mutation and screening, a total of 144 colonies were selected from the genetic library containing roughly 6 × 10^7^ mutants. After fermentation in 24-well plates, 58.3% (84/144) of the mutants produced higher VB_12_ levels than the initial strains (Fig. 6). Six mutants (*MD1–6, MB3–5, MD3–2 MC4–5, MB5–1,* and *MC5–2*) showed a significant enhancement of 10% in their VB_12_ content compared with the initial strains. These mutants were then cultured in 250 mL flasks at 30 °C with shaking for 7 days. The mutant MC*5–2* showed VB_12_ production of 156 ± 4.2 mg/L, which was 21.82% higher than the wild-type strain. After 7-generation cultivation, mutant *MC5–2* still maintained high VB_12_ yield reaching to 154 ± 3.2 mg/L. The VB_12_ titer of *MD3–2* was the second highest among the mutants with an increase of 20.31% than that of the wild-type strain.

### Whole genome sequencing analyses

To identify the gene mutations contributing to the high VB_12_ production, we sequenced the genomic DNA of the mutant *Sm320MC5–2*. The gene mutations were identified from the sequence data. A total of 17 SNP (single nucleotide polymorphism) and 1 Indel (insertion and deletion) were identified in the *Sm320MC5–2* genome (Additional file 1: Table S3). Genome-scale gene expression analysis of *Sm320* and *Sm320MC5–2* revealed that the mutant genes were located in the VB_12_ metabolic pathway, heme branch pathway, ABC transporters, VB_12_ precursor synthesis pathway, tricarboxylic acid cycle and other metabolic pathways. These SNPs functioned through altering the transcription level or enzyme activities and may have resulted in enhanced metabolic flux towards production of VB_12_ or by weakening the competing branches. This study demonstrates a new paradigm of revealing unexpected gene mutations to guide metabolic engineering for increasing VB_12_ yield.

## Discussion

In the past few years, various mRNA elements containing engineered riboswitches and reporter genes have been described and applied in modulating genetic switches, discovering the genetic pathway and optimizing cellular behaviors. However, none of simple and direct metabolite sensors has been applied in screening to increase the yield and productivity of target molecules. In this study, we reported successful application of a riboswitch sensor as a sensitive and novel detection method for VB_12_ screening. Moreover, we identified a VB_12_ riboswitch element that is responsible for expression of the VB_12_ transporter, *btuB*, in the genome of *Salmonella typhimurium* [29]. We validated the applicability of this riboswitch by screening higher VB_12_ producers from a genetic DNA library.

It is necessary to emphasize that the titer of VB_12_ was heavily dependent on the culture conditions. In our previous study, we did some research to select the nitrogen source, considering the sediment of CSL may interfere the accuracy of the experiments, we replaced the nitrogen sources “2% CSL” by “2% AYP, 1% peptone” in the fermentation medium when the strains were screened by FACS, and the comparison was shown in Additional file 1: Figure S5. When the strains were cultivated on shake flasks, VB_12_ was produced at millimolar concentrations. But when the strains were screened by FACS, the amount of intracellular VB_12_ was only about 1.06 μg/mL (micromolar levels of B_12_). So, the levels of VB_12_ produced could be detected by riboswitch normally.

In this study, 156 ± 4.2 mg/L of VB_12_ was produced by the selected mutant *MC5–2.* In order to identify gene mutations that contributed to the high production of VB_12_, we sequenced the genomic DNA of the mutant *Sm320MC5–2* Many gene mutations in *Sm320MC5–2* genome may play key roles in the VB_12_ hyperproduction. The SNPs in *hemin, hemN, sdhA, betB, ribH,* and *metH* were not reported in *S. meliloti*. These SNPs may alter transcription levels and enzyme activities to enhance metabolic flux towards producing VB_12_ or weakening the competing branches to improve productivity.

There were two changes identified in the heme branch pathway, mutation of *hemin* (c.C1228T: p.H410Y) and *hemN* (c.C632T: p.T211I). The mutation of *hemN* is speculated to decrease urinary porphyrins original III metabolic flow to the synthesis of hemoglobin leading to increasing productivity of VB_12_. The gene *hemin* encodes an iron complex transport system substrate-binding protein, and its mutation may decrease the active efflux of hemoglobin, reducing the heme anabolic flow to maintain the cell intracellular requirement.

In addition, we speculate that the mutation of *sdhA* (c.G1846A:p.A616T) in the tricarboxylic acid cycle conversion of succinate as carbon sources is necessary but insufficient for the VB_12_ metabolic pathways. The mutant of *betB* (c.T301C:p.W101R) gene may enhance the utilization of betaine to improve VB_12_ productivity. The *ribH* (c.T95C:p.L32P) encodes riboflavin kinase in riboflavin metabolism and may play a key role in 5, 6-dimethylbenzimidazo-le (DMB) synthesis, a precursor of VB_12_ biosynthesis.

Finally, one cobalamin-dependent enzyme in *S. meliloti*, methionine synthase (MetH c.G733A:p.G245S), catalyzes the final step in methionine biosynthesis and is required for growth [30]. In future work, the contribution of these enzymes to VB_12_ production needs to be determined, which may help elucidate the underlying mechanism and analyze metabolic circuits for further improvement of VB_12_ production in *S. meliloti*.

## Conclusions

We developed a Vitamin B_12_ high-throughput screening system by riboswitch sensor for the selection of VB_12_hyperproducing strains. The *S. meliloti* mutant library was generated by ARTP. Numerous mutants were obtained, and *Sm320MC5–2* exhibited 21.82% higher VB_12_ yield than the wild type strain. The genomic DNA of the hyperproducing mutant was subjected to whole genome sequencing, and the potential contributions of the gene mutations were discussed. This study provides a new paradigm in high throughput screening and elucidating the unexpected gene mutations which would be helpful for rewiring metabolic pathway to raise the yield of VB_12_.

## Additional file

Additional file 1: **Figure S1.** Growth conditions of *Sm320-N8*, *Sm320-L6 *and *Sm320* producing strains. **Figure S2.** Cloning scheme for riboswitch sensor constructs showing the relevant plasmid features. **Figure S3.** Characterization of our biosensor plasmids in response to addition of increasing VB_12_ into the medium. **Figure S4.** Lethal rate of *S. meliloti* irradiated by atmospheric and room temperature plasma (ARTP). **Figure S5.** Concentration of VB_12_ in producer strains during different growth conditions. **Table S1.** Oligonucleotide primers used in in this study. **Table S2.** Sequences of eight cobalamin riboswitches. **Table S3.** Analysis of mutations of mutant Sm320MC5–2 with original strain. Supplementary experimental procedures: VB_12_ feeding assay. (DOC 322 kb)
