# Supplementary material for: Engineering a vitamin B12 high-throughput screening system by riboswitch sensor in Sinorhizobium meliloti
Source: BMC Biotechnol. 2018 May 11;18:27. doi: 10.1186/s12896-018-0441-2 (PMC5948670; doi:10.1186/s12896-018-0441-2)
Supplement: Supplementary file 1 — Figure S1. Growth conditions of Sm320-N8, Sm320-L6 and Sm320 producing strains. Figure S2. Cloning scheme for riboswitch sensor constructs showing the relevant plasmid features. Figure S3. Characterization of our biosensor plasmids in response to addition of increasing VB12 into the medium. Figure S4. Lethal rate of S. meliloti irradiated by atmospheric and room temperature plasma (ARTP). Figure S5. Concentration of VB12 in producer strains during different growth conditions. Table S1. Oligonucleotide primers used in in this study. Table S2. Sequences of eight cobalamin riboswitches. Table S3. Analysis of mutations of mutant Sm320MC5–2 with original strain. Supplementary experimental procedures: VB12 feeding assay. (DOC 322 kb) [file 12896_2018_441_MOESM1_ESM.doc]

**Supplementary Data**

**Engineering a Vitamin B_12_ high-throughput screening system by riboswitch sensor in *Sinorhizobium meliloti***

Yingying Cai^1, 2, 3^ , Miaomiao Xia ^1, 2^ , Huina Dong ^1, 2^,Yuan Qian ^1, 2^, Tongcun Zhang^3^ , Beiwei Zhu^4^, Jinchuan Wu^5^, Dawei Zhang ^1, 2, 4^*

*Corresponding author: zhang_dw@tib.cas.cn, +86-22-24828749

Email address for all authors:

yy_cai@163.com; xia_mm@tib.cas.cn; dong_hn@tib.cas.cn; qian_yuan@tib.cas.cn;

tony@tust.edu.cn; zhubeiwei@163.com; wu_jinchuan@ices.a-star.edu.eg

^1^ Tianjin Institute of Industrial Biotechnology, Chinese Academy of Sciences, 32 West 7th Avenue, Tianjin Airport Economic Area, Tianjin 300308, China

^2^ Key Laboratory of Systems Microbial Biotechnology, Chinese Academy of Sciences, 32 West 7th Avenue, Tianjin Airport Economic Area, Tianjin 300308, China

^3^College of Biotechnology, Tianjin University of Science & Technology, No. 29, thirteenth Avenue Binhai District Tianjin, 300457, China

^4^ School of Food Science and Technology, Dalian Polytechnic University, National Engineering Research Center of Seafood, Dalian, 116034, P.R. China

^5^ Industrial Biotechnology Division, Institute of Chemical and Engineering Sciences, 1 Pesek Road, Jurong Island, Singapore 627833

**Supplementary Figures**

**
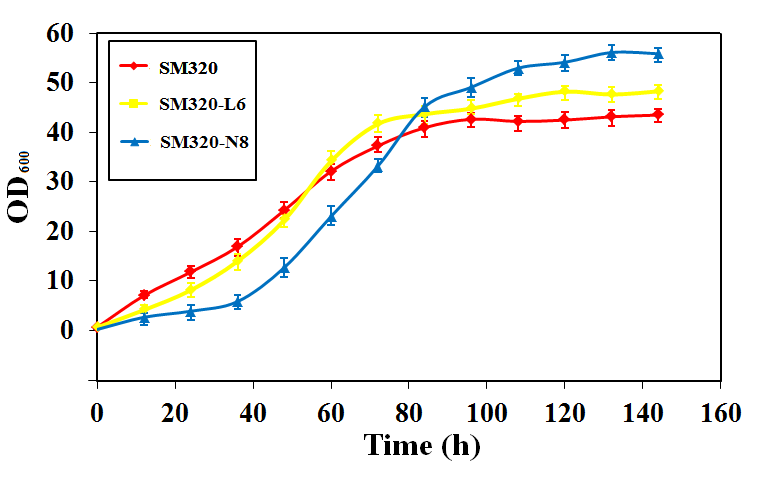
**

**Figure S1** Growth conditions of *Sm320-N8*, *Sm320-L6,* and *Sm320* producing strains. The fermentation of three strains were carried out in 250 mL shake flasks and samples were collected and measured within every 12 hours.


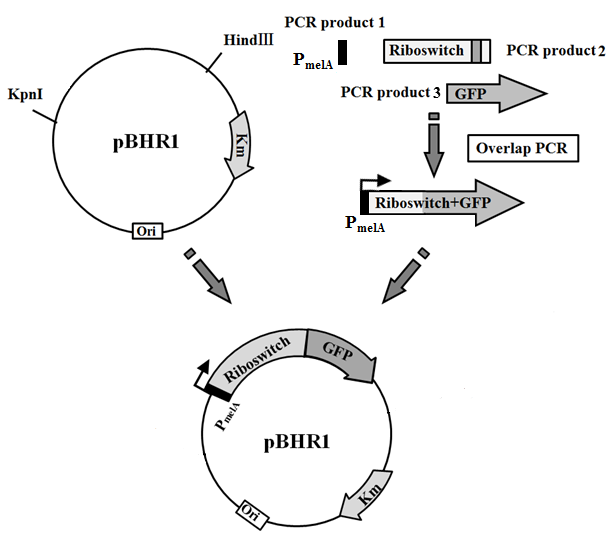


**Figure S2** Cloning scheme for riboswitch sensor constructs showing the relevant plasmid features. Genetic maps of pBHR1, the light gray shaded area included in the insert riboswitch was obtained by PCR amplification of the leader mRNA. Details of riboswitch sequences are provided in Supplementary Table.

**
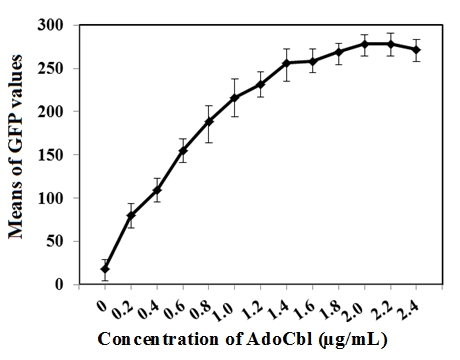
**

**Figure S3** Characterization of our biosensor plasmids in response to addition of increasingVB_12_ into the medium. A corresponding rise in GFP fluorescence values of *Sm320-N8* strains carrying SY-btuB sensor double plasmid, and we were able to show that the respond range of this riboswitch was approximately 0.01~1.8 µg/mL AdoCbl.

**
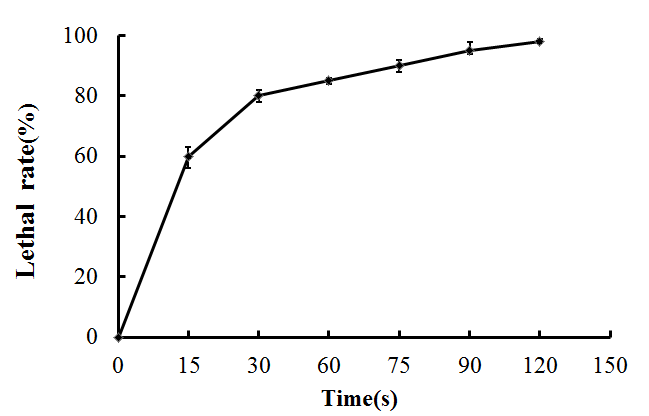
**

**Figure S4** The lethal rate of *S.* *meliloti* irradiated by atmospheric and room temperature plasma (ARTP).The line was the lethal rate of *S. meliloti* sampled treated by ARTP with a radio-frequency (RF) power input of 100 W for 15, 30, 60, 75, 90, and 120 s. The irradiation conditions: treated for 60 s, RF power input of 100 W, helium gas flow rate of 10 L/min, distance between the *S. meliloti* cells and the plasma source of 4 mm.

**
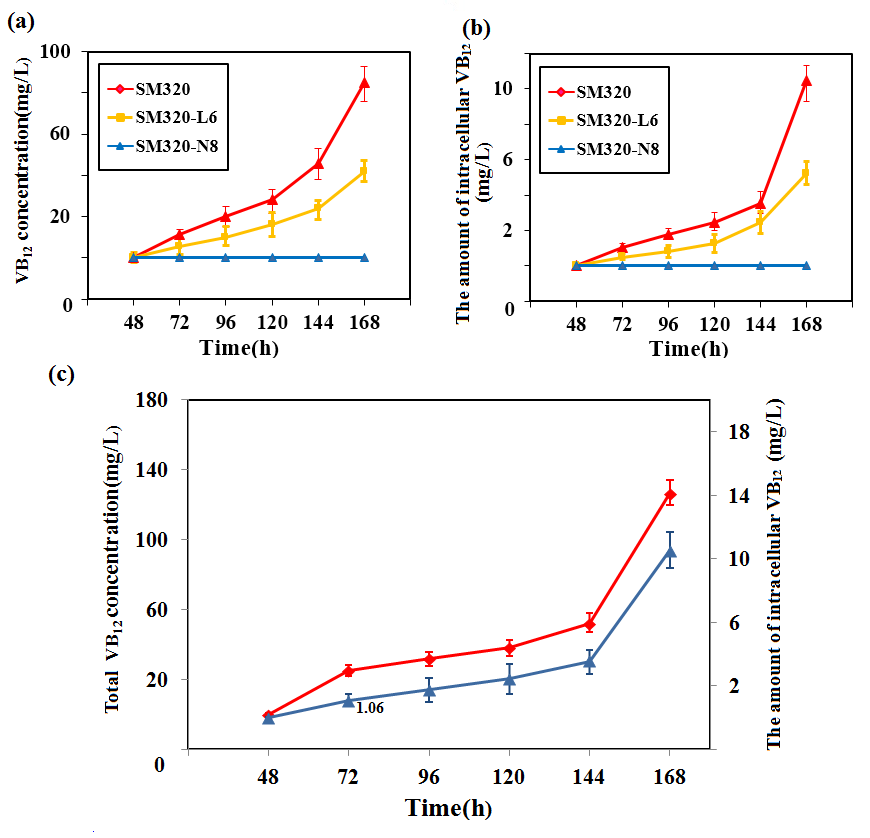
**

**Figure S5** The concentration of VB_12_ in producer strains during different growth conditions. (a) The concentration of VB_12_ in producer strains in “2%AYP, 1% peptone” medium fermentation broth was determined by high performance liquid chromatography (HPLC). Different colors represent the different strains. Values shown are HPLC-determined and are the means of three independent fermentation cultures for each strain. (b) The amount of intracellular VB_12_ concentration in recombinant strains. (c) The red rhombus meant total concentration of VB_12_ in shake flask fermentation, the blue triangles meant the amount of intracellular VB_12_ concentration when we screen hyperproducing strain by FACS.

**Supplementary Table**

**Table S1.** Oligonucleotide primers used in in this study

| Primer | Sequence 5’→3’ |
| --- | --- |
| cobI _up_F | GCTGATCGGATTGTAGAGGGCAATG |
| cobI _up_R | CCGGTACGCTCTATGTCGTCGGTAC |
| cobI _down_F | AGGCCCGGCCTCGCGAGCGA |
| cobI_down_R | GCGACCAGGCAGTTCGTGTTT |
| lacI-F | CGTGGATTTGAGGTTCTTGTCCGTGAAACCAGTAACGTTATACGAT |
| lacI-R | TGAATCCGTAATCATGGTCATAGC |
| gfp-F | ATGAGTAAAGGAGAAGAACTT |
| gfp-R | TTATTTGTATAGTTCATCCATG |

**Table S2.**Sequences of eight cobalamin riboswitches

| . **Sensor name** | **Sensor sequence** |
| --- | --- |
| EC-butB | CCACTTGCCGGTCCTGTGAGTTAATAGGGAATCCAGTGCGAATCTGGAGCTGACGCGCAGCGGTAAGGAAAGGTGCGATGATTGCGTTATGCGGACACTGCCATTCGGTGGGAAGTCATCATCTCTTAGTATCTTAGATACCCCTCCAAGCCCGAAGACCTGCCGGCCAACGTCGCATCTGGTTCTCATCATCGCGTAATATTGATGAAACCTGCGGCATCCTTCTTCTATTGTGGATGCTTTACA |
| PF-cbiB | TGTACTAGGGTCAATGTGCTGGCCTCGGCCAGCGCGCGTCCGCAGCGAAGCCGGTGGGAATCCGGCACTGTCCCGCAACGGTGATGGGGCCCGGCCCCGAGTCCGGTCGACTGTGGACGTGTCCCCACATTGCCCCGGCGCAAGGTCAGCCCCACGAGCTGCCTGCGCGTGCACCGCGAAGGACGGCCCGTGGATTTGAGGTTCTTGTCCGAG |
| SM-bluB | TGCCGCCGTCAGGTGCCCGCGAAATGCGGGGGAATCGGGAAGCCGGTGCAGTTCCGGCACGTGCCCAACGCTGTGAAGGGGACGTTCTCGCCAAAAAGGGCTCTGAATCTTTTCAGAGCTTTGCCACTGAATATTGAAGCTATTCGGGAAGGCGGCGCGAACGGATGATCCGAAGTCAGAAGACCGGCCTGGCGAGATAGACCGGCCCCGCGCGGACGGCGGGAGGTTTCCGCATTGTTGGGGATCGAACG |
| SM-cobU | GCCGGGCAGGACGCCTCTTCTTCTACGAATCGTCCGCCTTTCGCGATGCCGCAAACGCCGGGAAGGCGAGGCGAGCCCGTTCGGTCTTTTGCCGCATCGTTTTTCGGGCCGAGCCGGTCCGGCGAACGTGCGGCCATGAGGATCGTGACGCCGTGAGCCAGGAGACCTGCCATCCGTCAGGGCATTCCGCCCGAGGGGAGATGCTCCCCGATGCCAGCACGGAGGTT |
| MLO-bluB | CGCCACTGCCTGGTGCCCGCCGCAAGCGGGAGAATCGGGAACACGGTTGAACTCCGTGGCGTGCCCAACGCTGTAAGGGGGACCGCGCCGGTAAATGCCACTGTCGATGACGGGAAGGCACCGGACGCGGGTTGATCCCGAGCCAGAAGACCGGCCTGGCAGGCATCGTCATCCGCATGGTCAGGCGGACGACATTGATTGCAATCGCAAGGGGACGAGCGATGCCGGAACAC |
| MLO-metE | GCGCATGTCGTGGTTCTCCGCGCGGCACTGCCGTAGCGGAGCTAAGAGGGAAGCCGGTGCGATGCCGGCGCTGCCCCCGCAACTGTTAGCGGCGAGCCAAGCCCATTGGTGTCACTGAGGCGAACGGCCTCGGGAAGACGGGCAGAGGCTTTGACCCGCGAGCCAGGAGACCTGCCACGACGAACAACGTCCACGGGCGGGGTGTCTCGGTGGCCGCGGTAGCCTGGCTTCGTGCCGCCTGCTCGCGCGTTCCTTGTCCCCACGCCCCAACCATCGGGGTATGGCATGACTGTCTCTCAACAAATTCCA |
| SY-btuB | ATCCGTGGGCCGGTCCTGTGAGTTAATAGGGAATCCAGTGAAAATCTGGAGCTGACGCGCAGCGGTAAGGAAAGGTGAGATGAGAGCGTAAGCAGACACTGCCTCCGGCGGGAAGTCATCATTTCTGCTATCCAGCCAACGGATAACCCTCCAAGCCCGAAGACCTGCCGGCTAACGTCGCATCTGGTTTTTCATCATCGCGTACTATCGATGAAGCCTGCGGCATCCTTCTTATATTGTGGATGCTTTACA |
| BI-cbiW | CAGTAAAGATGCCAAGAAGGCTTAATAGGGAAACTGGTGAAAGACCAGTACTGCCCCCGCAACTGTAAGTGTGGACGAACGAGTATAACCACTGTGGAAAAATCACGGGAAGGTTCTCAAGTAGAATGATACACAAGTCAGGAGACCTGTCTTTATTGTGAAGTTTCTATTTCTCCGGGGAGCGGGAAGGGAAACGTTGGCTTAAAGTGGCATTCGTGTACACTCTTATTCTGACGTTTTACCCGCTCGCATATTAGCGAAGCGGG |

The sequence of the riboswitches that cnserved RNA elements upstream of some B12-regulated genes

**Table S3. Analysis the mutations of the Sm320MC5-2 mutant with original strain**

| Gene | Variation | | Gene Function | | Pathway Involved | |  | | |
| --- | --- | --- | --- | --- | --- | --- | --- | --- | --- |
| SNP (single nucleotide polymorphism) | | | | | | |  | | |
| *hemD*  (orf3362) | | Exonic：c^a^.C158T:p^b^.A53V | uroporphyrinogen III synthase | | | VB_12_ metabolic pathway |  | | |
| *cobL*  orf3188 | | Exonic：c.A788G:p.E263G | precorrin-6Y C5,15-methyltransferase (decarboxylating) [EC:2.1.1.132] | | | VB_12_ metabolic pathway |  | | |
| *cobI-cbiL*  orf3191 | | Exonic：c.A310G:p.T104A | precorrin-2/cobalt-factor-2 C20-methyltransferase [EC:2.1.1.130 2.1.1.151] | | | VB_12_ metabolic pathway |  | | |
| *cobQ*  orf1811 | | Exonic：c.G513A:p.T171T | adenosylcobyric acid synthase [EC:6.3.5.10] | | | VB_12_ metabolic pathway |  | | |
| *metH*  orf1820 | | Exonic：c.G733A:p.G245S | 5-methyltetrahydrofolate--homocysteine methyltransferase [EC:2.1.1.13] | | | Methionine biosynthesis |  | | |
| *acrB*  orf2221 | | Exonic：c.A2129G:p.D710G | membrane fusion protein | | | Efflux pump |  | | |
| *acrA*  orf2222 | | Exonic：c.C149T:p.A50V | membrane fusion protein | | | Efflux pump |  | | |
| *hemN*  orf5058 | | Exonic：c.C1228T:p.H410Y | oxygen-independent coproporphyrinogen III oxidase [EC:1.3.99.22] | | | Heme branch pathway |  | | |
| *hemin*  orf2706 | | Exonic：c.C632T:p.T211I | iron complex transport system substrate-binding protein | | | Heme branch pathway |  | | |
| *sdhA*  orf3352 | | Exonic：c.G1846A:p.A616T | succinate dehydrogenase flavoprotein subunit [EC:1.3.99.1] | | | Tricarboxylic acid cycle |  | | |
| *PC*  orf3515 | | Exonic： c.C1507T:p.P503S | pyruvate carboxylase [EC:6.4.1.1] | | | Tricarboxylic acid cycle |  | | |
| *betB*  orf3256 | | Exonic： c.T301C:p.W101R | betaine-aldehyde dehydrogenase [EC:1.2.1.8] | | | Betaine metabolic pathway |  | | |
| *opuC* orf5633 | | Exonic：c.C262T:p.L88F | osmoprotectant transport system substrate-binding protein | | | ABC transporters |  | | |
| *gsiA*  orf4151 | | Exonic：c.T431C:p.M144T | glutathione transport system ATP-binding protein | | | ABC transporters |  | | |
| *potD*  orf4281 | | Exonic：c.T302C:p.M101T | spermidine/putrescine transport system substrate-binding protein | | | ABC transporters |  | | |
| *tauC*  orf6654 | | Exonic：c.C643T:p.L215F | taurine transport system permease protein | | | ABC transporters |  | | |
| *paaH*  orf2882 | | Exonic：c.T746C:p.V249A | 3-hydroxybutyryl-CoA dehydrogenase [EC:1.1.1.157] | | | Phenylalanine metabolism |  | | |
| Indel (insertion and deletion) | | | | | | |  |  |  |
| *doeC*  orf5767 | Exonic：c.12_13insG;p.G4fs | | aspartate-semialdehyde dehydrogenase [EC:1.2.1.-] | Glycine, serine and threonine metabolism | | |  | | |

^a^ The position of the gene on chromosome

^b^ The position of the gene on peptide chain

^c^ The genome sequence of strain *Sinorhizobium meliloti* 320 is available from GenBank with Accession No. POVM00000000

**Supplementary experimental procedures**

**VB_12_ feeding assay**

A single colony of *Sm320-N8* with SY-btuB sensor double plasmid was inoculated into LB/MC medium and grown at 30°C overnight. Cells were washed twice by PBS and 10^6^ bacteria were inoculated into fresh M9/sucrose medium in 24-well plates supplemented with 0.01~5 mg/L of Ado-Cbl. Cells were collected in the next 5h and resuspended in cold PBS solution. The fluorescence value was measured by FCM (flow cytometry, MoFlo XDP, Beckman, USA) and used to detect intracellular VB_12_ concentration as described below.
